# Global control of COVID-19: good vaccines may not suffice

**DOI:** 10.26633/RPSP.2021.148

**Published:** 2021-12-10

**Authors:** Javier Eslava-Schmalbach, Eric B. Rosero, Nathaly Garzón-Orjuela

**Affiliations:** 1 Universidad Nacional de Colombia Bogotá Colombia Universidad Nacional de Colombia, Bogotá, Colombia; 2 University of Texas Southwestern Medical Center Dallas Texas United States of America University of Texas Southwestern Medical Center, Dallas, Texas, United States of America

**Keywords:** Coronavirus infections, COVID-19, SARS-CoV-2, COVID-19 vaccines, immunity, herd, vaccination, health equity, Infecciones por coronavirus, COVID-19, SARS-CoV-2, vacunas contra la COVID-19, inmunidad colectiva, vacunación, equidad en salud, Infecções por coronavirus, COVID-19, SARS-CoV-2, vacinas contra COVID-19, imunidade coletiva, vacinação, equidade em saúde

## Abstract

The COVID-19 pandemic has unveiled health and socioeconomic inequities around the globe. Effective epidemic control requires the achievement of herd immunity, where susceptible individuals are conferred indirect protection by being surrounded by immunized individuals. The proportion of people that need to be vaccinated to obtain herd immunity is determined through the herd immunity threshold. However, the number of susceptible individuals and the opportunities for contact between infectious and susceptible individuals influence the progress of an epidemic. Thus, in addition to vaccination, control of a pandemic may be difficult or impossible to achieve without other public health measures, including wearing face masks and social distancing. This article discusses the factors that may contribute to herd immunity and control of COVID-19 through the availability of effective vaccines and describes how vaccine effectiveness in the community may be lower than that expected. It also discusses how pandemic control in some countries and populations may face vaccine accessibility barriers if market forces strongly regulate the new technologies available, according to the inverse care law.

## RESUMEN

La pandemia de COVID-19 ha puesto al descubierto inequidades socioeconómicas y de salud en todo el mundo. Un control epidémico eficaz requiere el logro de la inmunidad colectiva, mediante la cual se confiere a las personas vulnerables una protección indirecta al estar rodeadas de personas inmunizadas. El umbral de inmunidad colectiva determina la proporción de personas que deben vacunarse para llegar a la inmunidad colectiva. Sin embargo, el número de personas vulnerables y las oportunidades de contacto entre las personas infecciosas y las personas vulnerables influyen en el progreso de una epidemia. Por lo tanto, además de la vacunación, el control de una pandemia puede ser difícil o imposible de lograr sin otras medidas de salud pública, como las mascarillas y el distanciamiento social. Este artículo trata sobre los factores que pueden contribuir al logro de la inmunidad colectiva y el control de la COVID-19 mediante la disponibilidad de vacunas efectivas y describe cómo la efectividad de las vacunas en la comunidad puede ser inferior a la prevista. También aborda cómo el control pandémico en algunos países y grupos poblacionales puede enfrentarse a obstáculos que dificultan la accesibilidad de las vacunas si las fuerzas del mercado son el principal factor que regula las nuevas tecnologías disponibles, como se indica en la ley de atención inversa.

## RESUMO

A pandemia de COVID-19 revelou iniquidades socioeconômicas e de saúde no mundo todo. Um controle epidêmico eficaz requer a obtenção da imunidade coletiva, em que indivíduos suscetíveis recebem proteção indireta por estarem rodeados de indivíduos imunizados. A proporção de pessoas que precisam ser vacinadas para se alcançar a imunidade coletiva é definida pelo limiar da imunidade coletiva. Porém, o número de indivíduos suscetíveis e as oportunidades de contato entre indivíduos infecciosos e suscetíveis influenciam o progresso de uma epidemia. Portanto, além da vacinação, o controle de uma pandemia pode ser difícil ou impossível de ser alcançado sem outras medidas de saúde pública, incluindo o uso de máscaras e o distanciamento social. Este artigo discute os fatores que podem contribuir para a imunidade coletiva e para o controle da COVID-19 por meio da disponibilidade de vacinas eficazes, e descreve como a eficácia das vacinas na comunidade pode ser menor do que o esperado. Também discute como o controle da pandemia em alguns países e populações pode enfrentar barreiras de acessibilidade às vacinas se as forças de mercado regularem fortemente as novas tecnologias disponíveis, conforme a lei dos cuidados inversos.

The current COVID-19 pandemic caused by SARS-CoV-2 has unveiled preexisting socioeconomic and health care inequities between and within countries worldwide, with higher morbidity and mortality in well-known disadvantaged populations (1, 2). The approval of recently developed COVID-19 vaccines (3, 4) has raised expectations that the pandemic may soon be controlled in most parts of the world, similar to what has been achieved worldwide through vaccination programs for other outbreaks such as smallpox, polio, measles, and Ebola virus disease (5–9). However, availability of vaccines is only one component of the complex process needed for pandemic control. Minimization of epidemic spread requires the creation of immunization programs, preceded by identification of high-risk populations, aimed at reaching and maximizing immunization of subgroups with highest susceptibility to the disease (10).

To be highly successful, designing such programs requires a full understanding of the concept of herd immunity and of the factors that may influence effectiveness of the vaccine in the community. The reproduction number (i.e., a measure of the transmission potential of a disease), the original efficacy of the vaccines in clinical trials, population and provider adherence to the vaccination program, and the rate of natural immunity in the community are important concepts that need consideration. As the progress of an epidemic is not only influenced by the number of susceptible individuals but also by the opportunities for contact between infectious and susceptible individuals (11), in addition to vaccination, control of the COVID-19 pandemic may be difficult or impossible without continuous use of other public health measures, including the wearing of face masks, hand-washing, and social distancing.

Furthermore, pandemic control in some countries and populations may face the barrier of vaccine accessibility. In the case of COVID-19, where new vaccines (3) are in the process of being deployed in the community, there is the possibility of occurrence of an inequality phenomenon known as the inverse care law (12). In these circumstances, the vaccines would be available first to wealthier countries and, within countries, to groups with higher socioeconomic status, instead of being available first to the populations in most need. In this article, we discuss the factors that may contribute to herd immunity and control of the COVID-19 pandemic in the context of availability of recently developed vaccines.

## HERD IMMUNITY

The term herd immunity was first mentioned by Potter in 1918 in a descriptive study on contagious abortion in cattle (13). In 1923, Topley and Wilson (14) investigated herd immunity in experiments where they assessed the specific mortality from exposure to *Bacterium enteritidis* in various clusters of mice that differed in the proportions of immunized and non-immunized animals. The specific mortality rate in cages containing non-immunized mice was 96.7% compared with about 33.3% in those containing immunized mice. However, in cages where 1/3 of the animals were vaccinated, the mortality rate was much lower (56.7%) than that expected based on an individual immunity effect. The authors suggested that the “immunity of a herd” and individual immunity are different entities affected by various factors (14). Herd immunity can be understood then as the group resistance to the attack by an infectious disease given by a large proportion of immunized individuals that hinders the contact of susceptible individuals with those that are infective (10). Herd immunity is also referred to as indirect protection given by living in a highly immunized population (15).

In the 1950s and 1960s, herd immunity became a crucial concept for public health policy when using new vaccines (10). This proposal was used as a successful World Health Organization (WHO) strategy to eradicate smallpox in the world (7). The smallpox vaccination strategy determined that 80% of people needed to be vaccinated as the standard aim to obtain herd immunity (7). The vaccination coverage required to prevent outbreaks of infectious diseases in the population can be calculated based on the concept of the herd immunity threshold (It) (16, 17), defined as the proportion of individuals in a population who cannot transmit the disease because they have acquired immunity (17). Control of outbreaks of the disease will occur when the proportion of immune individuals in a community is larger than this threshold. A basic way of calculating It involves the use of the basic reproduction number (R0) (Equation 1). R0 represents the number of secondary infections produced by each infected individual in a completely susceptible population (18).


(Equation 1) (16, 17) $$\mathrm{It}=1-\left(\frac{1}{R0}\right)$$

During the evolution of a pandemic, this number changes because of the increasing number of naturally immunized people and different adherence levels to epidemiologic control measures, which is then called the current reproduction number, Rt (R at time t). The vaccination coverage (Vc)—i.e., the proportion of the population that needs to receive the vaccine to obtain herd immunity—can be calculated from the herd immunity threshold and the clinical vaccine efficacy (E) (Equation 2).


(Equation 2) (16, 17) $$\mathrm{Vc}=\frac{\mathrm{It}}{E}=\frac{\left(\left[1-\left(\frac{1}{\mathrm{Rt}}\right)\right]\right)}{E}$$

However, when a variable proportion of the population is partially protected by natural immunization (In), the vaccination coverage required to achieve herd immunity needs to be recalculated according to Equation 3.


(Equation 3) (16, 17) $$\mathrm{Vc}=\frac{It-In}{E}=\frac{\left[1-\left(\frac{1}{\mathrm{Rt}}\right)\right]-In}{E}$$

Based on the previous equations (16, 19), we constructed graphs to estimate the critical vaccination coverage required to obtain herd immunity using current data on the COVID-19 pandemic. We assumed that at the time the vaccines will be widely available to the public, about 10% to 20% of the population will be naturally immunized by exposure to the infection. A series of current reproduction numbers often reported for the COVID-19 pandemic (Rt from 1.5 to 6.0) (20, 21) were used for our estimates. This allows the introduction of variability on the pandemic’s behavior in the equations, given by a multi-intervention strategy that may include a combination of vaccination and other control measures (20, 21). A clinical efficacy of the COVID-19 vaccine of 90% (representing behavior in the controlled environment of clinical trials) was used for our estimates. A critical factor that impacts the success of vaccination programs in achieving herd immunity is the actual effectiveness of the vaccine in the community (Ec) (Equation 4). Ec is influenced by a variety of factors, including the immunological efficacy of the vaccine (E) (as reported in clinical trials), emergence of new variants of the virus against which the vaccine is less effective, population adherence to vaccination (ADHPo) (individuals may be willing to receive none, part, or the entire vaccination schedule), and provider adherence to the program (ADHProv) (maintenance of adequate vaccine cold chain, appropriate vaccine injection technique, effective follow-up of individuals to guarantee completion of the vaccination schedule). In high-income countries, Ec is probably not strongly affected by provider adherence but may be affected by population adherence in some communities and those with stronger antivaccine groups. In contrast, in low-income countries, Ec may be impacted both by population and provider adherence.


(Equation 4), adapted from (22, 23) $\mathrm{Ec}=E^{*}\mathrm{ADHpro}v^{*}\mathrm{ADHpo}$

Finally, Equation 5 includes all the above-mentioned factors used in the calculation of the critical vaccination coverage needed to obtain herd immunity.


(Equation 5) $$\mathrm{Vc}=\frac{\mathrm{It}-\mathrm{In}}{\mathrm{Ec}}=\frac{\left[1-\left(\frac{1}{\mathrm{Rt}}\right)\right]-\mathrm{In}}{\mathrm{Ec}}$$

**FIGURE 1.**
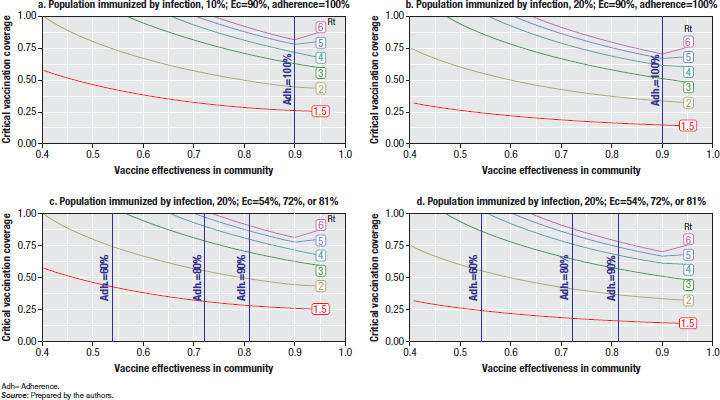
Vaccination coverage required to extinguish a COVID-19 outbreak in communities with various levels of adherence to vaccination programs and different degrees of natural immunization across a series of effective reproduction number values

Figures 1a and 1b display the critical vaccination coverage needed to establish herd immunity in a population with 10% or 20% rates of natural immunization by infection, respectively, in the ideal circumstance of 100% population and provider adherence. In this case, the vaccine effectiveness in the community would be 90%, equivalent to the 90% original clinical efficacy of the vaccine in the research trials. Herd immunity in these scenarios could be achieved by vaccination of a reasonable proportion of the population across a variety of Rt values. For example, for an Rt = 3.0, a 90% effective COVID-19 vaccine would require a critical vaccination coverage of 62% to obtain herd immunity when 10% of the population is naturally immunized (Figure 1a), and 51% when 20% of the population is naturally immunized (Figure 1b).

Adh= Adherence.

***Source***: Prepared by the authors.

The critical vaccination coverage required for herd immunity at different levels of vaccine effectiveness in the community (decreasing population or provider adherence) is displayed in Figures 1c and 1d. For any value of Rt, the proportion of the population that needs to be effectively vaccinated would increase exponentially as the adherence to the vaccination program decreases. Depending on variations on Rt and adherence, in some cases, even vaccination of the whole population would not suffice to extinguish the epidemic outbreak. For example, when the adherence to the vaccine is 60%, the respective effectiveness in the community is about 54% (Figure 1c). In this case, when Rt is equal to 2.0, about 75% of the population would need to be vaccinated to control a COVID-19 outbreak. However, if the Rt value increases to 3.0, even vaccination of 100% of the population eligible for vaccination would not be enough to control the outbreak by vaccination alone.

The spread of the virus depends on the number of susceptible individuals (which may be decreased by vaccination) but also on changes in the transmission rate (affected by other mitigation strategies) (24). Evidence on the efficacy of current COVID-19 vaccines to prevent viral transmission is scarce, and the extent of natural immunity conferred by the disease is uncertain. Therefore, it is of paramount importance to note that herd immunity may be impossible to achieve with vaccination alone (25, 26) in communities with low rates of adherence to vaccination programs (where the proportion of susceptible population remains high) and low rates of compliance with non-immunologic mitigation measures (which increases Rt due to higher chances of contact between infected and susceptible individuals). Despite the availability of highly effective vaccines, other control measures—including social distancing, use of face masks, and frequent hand-washing—should not be overlooked and are still necessary to obtain rapid control and eradication of the current COVID-19 pandemic (27–29). Furthermore, herd immunity works within the herd. If individuals move from or to other clusters, they will not be protected by the herd, which highlights the importance of minimizing movement of individuals between communities and/or the importance of achieving herd immunity everywhere. The emergence of new variants of SARS-CoV-2 that are not neutralized by preexisting antibodies or are less effectively covered by current vaccines has an additional effect on herd immunity. Also, vaccination programs initially exclude the population under 16 years of age, which may become a reservoir contributing to maintaining circulation of the virus in the community, and thus affecting the herd immunity threshold (30–32).

## INVERSE EQUITY AND THE INVERSE CARE LAW

As vaccines are the main component to achieve the herd immunity threshold in a population, the success of a systematic immunization program requires that vaccines reach all types of subgroups of the population, regardless of their socioeconomic condition, rather than aiming to reach any specified overall proportion of the population. As the COVID-19 pandemic has spread worldwide, demand has exceeded supply of the vaccines. Hart proposed the inverse care law in 1971, which happens when “the availability of good medical care tends to vary inversely with the need of the population served” (12). In other words, the implementation of new medical technologies is pushed by market forces to a population with less need instead of people who need it the most. Furthermore, Hart theorized that the inverse care law operated entirely when medical care was exposed to market forces (12). This law was later validated by Victora et al. (33) as inverse equity, showing how inequity ratios increased between rich and poor people, based on morbidity and mortality indicators, when a new public health intervention was implemented.

**FIGURE 2.**
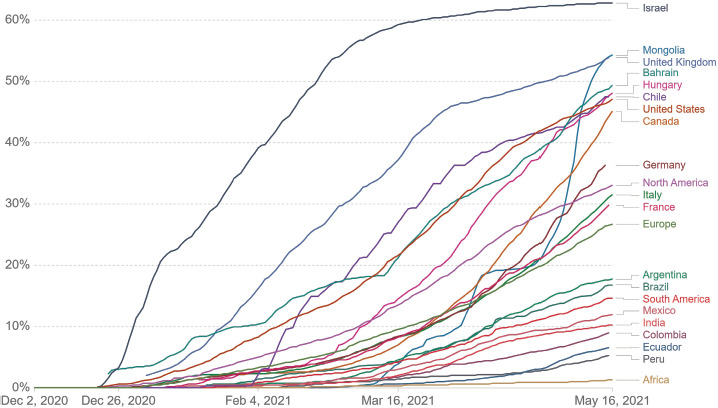
Coverage of COVID-19 vaccination: people who received at least one dose of the vaccine

***Source***: Ritchie H, Ortiz-Ospina E, Beltekian D, Mathieu E, Hasell J, Macdonald B, et al. Coronavirus (COVID-19) Vaccinations - Statistics and Research [Internet]. Our World in Data (open source). 2021 [cited 2021 May 17]. Available from: https://ourworldindata.org/covid-vaccinations

In the case of the COVID-19 pandemic, the vaccine is the new public health intervention that has been introduced in the world as a new “business.” Market forces have pushed this technology to the wealthiest countries and the most affluent people within the nations (34) (Figure 2). In 2020, several high-income nations created stimulus packages in the form of grants and loans to promote the development of COVID-19 vaccines. In return, vaccine developers entered into agreements that gave their sponsors priority access to future vaccines (35). As a result, high-income countries, which comprise 14% of the world’s population, had purchased 53% of the annual production of COVID-19 vaccines and had vaccinated about 25% of their populations by the first trimester of 2021. In contrast, vaccination had reached less than 1% of people living in low-income countries (36–38). Considering the Americas, of the 380 million vaccine doses administered worldwide, 29% have been given to the 331 million population of the United States of America and only 6% to the 431 million population of South America. The COVID-19 Vaccines Global Access (COVAX) facility emerged as a global solidarity effort aimed at narrowing vaccine access inequalities by vaccinating high-risk populations in both high- and low-income countries (38, 39). However, vaccine-hoarding actions of rich countries have diminished the ability of COVAX to provide a sufficient amount of vaccines to low- and middle-income countries. Accordingly, it is predicted that <20% of the population of these countries will be fully vaccinated by the end of 2021. Furthermore, because of the lack of capacity to appropriately refrigerate and administer the vaccine, current COVID-19 vaccines may take even longer to reach remote parts of low-income countries. Finally, evidence of racial and socioeconomic inequalities in the distribution of COVID-19 vaccines and the effects of this have been revealed within various nations, where high-risk people in communities of low-income status, racial and ethnic minorities, and persons with disability are having less access to the vaccines (40–43). It appears that the inverse equity law has been proved again in the context of the new COVID-19 vaccines, and hence, the world will need several years to control the pandemic (34) (Figure 2).

In conclusion, effective control of an epidemic outbreak requires achievement of herd immunity, where susceptible individuals receive indirect protection by living in a population that is highly immunized, either by natural immunization from infection or by vaccination. The proportion of the population that requires vaccination to obtain herd immunity can be determined by the calculation of the herd immunity threshold. However, the required vaccination coverage is affected by the proportion of naturally immunized individuals in the population and by factors that may decrease the effectiveness of the vaccine compared with what was observed in clinical trials. Based on current epidemiologic data on the COVID-19 pandemic, we provided some examples of the vaccination coverage required to extinguish a COVID-19 outbreak in communities with various levels of adherence to vaccination programs and different degrees of natural immunization. As the spread of infectious disease depends also on the opportunities for contact between infected and susceptible individuals, in communities with high disease transmission potential (large R0) and low adherence to vaccination, herd immunity and outbreak control may not be obtainable even if the entire population eligible for vaccination receives the vaccine. Therefore, other epidemiologic control measures, including social distancing, use of face masks, and frequent hand-washing, should not be overlooked. Low- and middle-income countries face additional barriers to pandemic control. By the effects of the inverse equity law, market forces have made most of the initial production of the newly developed COVID-19 vaccines available to wealthier nations first. Low- and middle-income countries have been suffering and will keep suffering the prolonged morbidity and mortality associated with the pandemic. It is highly relevant to monitor and research emerging inequalities related with COVID-19 vaccine coverage as a result of the inverse care law.

## Disclaimer.

Authors hold sole responsibility for the views expressed in the manuscript, which may not necessarily reflect the opinion or policy of the *RPSP/PAJPH* and/or the Pan American Health Organization.
